# Treatment patterns and outcomes according to cytogenetic risk stratification in patients with multiple myeloma: a real-world analysis

**DOI:** 10.1038/s41408-022-00638-0

**Published:** 2022-03-23

**Authors:** Shebli Atrash, Evelyn M. Flahavan, Tao Xu, Esprit Ma, Sudeep Karve, Wan-Jen Hong, Gilbert Jirau-Lucca, Michael Nixon, Sikander Ailawadhi

**Affiliations:** 1 Levine Cancer Institute-Morehead (Hematology), Charlotte, NC USA; 2 Roche Products Ltd, Welwyn Garden City, UK; 3 F. Hoffmann-La Roche Ltd, Basel, Switzerland; 4 Genentech, Inc., South San Francisco, CA USA; 5 AbbVie, Inc., North Chicago, IL USA; 6 Mayo Clinic, Jacksonville, FL USA; 7 Present Address: Imago BioSciences, South San Francisco, CA USA

**Keywords:** Cancer, Risk factors

## Abstract

A clearer understanding of the prognostic implications of t(11;14) in multiple myeloma (MM) is needed to inform current and future therapeutic options. We utilized real-world data from a US database to examine treatment patterns and outcomes in patients by t(11;14) status compared with high- and standard-risk subgroups across different lines of therapy (LoT). This retrospective, observational cohort study used de-identified patient-level information from adults with MM and first-line treatment initiation between January 2011 and January 2020, followed until February 2020. The high-risk cohort comprised patients with high-risk genetic abnormalities per mSMART criteria (including those with co-occurring t(11;14)). Among 6138 eligible patients, 6137, 3160, and 1654 received first-, second-, and third-line treatments, respectively. Of 645 patients who had t(11;14), 69.1% had t(11;14) alone, while 30.9% had co-occurring high-risk abnormalities. Altogether, 1624 and 2544 patients were classified as high- and standard-risk, respectively. In the absence of biomarker-driven therapy, treatment patterns remain similar across LoT in high-risk, t(11;14)+, and standard-risk subgroups. Across all LoT, patient outcomes in the high-risk subgroup were less favorable than those in the t(11;14)+ and standard-risk subgroups. Thus, there is an opportunity for novel therapeutics targeted to t(11;14) and other defined subgroups to personalize MM therapy and optimize patient outcomes.

## Introduction

Multiple myeloma (MM) is an incurable and heterogeneous disease, with a 5-year overall survival (OS) of approximately 55% [1, 2]. Induction therapies for MM consist of doublet or triplet combinations of agents with different mechanisms of action including corticosteroids (e.g., dexamethasone), immunomodulatory drugs (IMiDs; e.g., lenalidomide), and proteasome inhibitors (PIs; e.g., bortezomib). A triplet combination including bortezomib and dexamethasone (e.g., bortezomib, lenalidomide, and dexamethasone [VRd]) has been the standard-of-care for first-line induction in newly diagnosed patients [3]. Recommendations for subsequent lines of therapy suggest the use of regimens that the patient had not previously been exposed to.

The MM treatment landscape has evolved significantly during the last decade, with the use of triplet combinations increasing and doublet combinations decreasing, and a trend toward longer OS has been reported [4]. Survival outcomes are affected by several factors, including patient characteristics such as age and fitness, race, tumor burden, and genetic abnormalities involving chromosomes 14, 1p, 1q, 13, or 17 [5–12]. Several risk subgroups of patients with MM can be defined based on these cytogenetic abnormalities.

The chromosome translocation t(11;14), which results in dysregulation of cyclin D1 and increased cell cycle progression [13, 14], occurs in ~16% of patients with MM (observed in trials from the Eastern Cooperative Oncology Group [ECOG] and the Intergroup Francophone du Myélome [IFM]) [14–16], and has historically been considered to be a standard-risk chromosomal abnormality [17, 18]. Some recent studies, however, have reported findings suggesting that prognosis may be poorer than previously expected in patients with t(11;14) compared with patients otherwise classified as having standard-risk MM [19–21]. There is also a lack of available data on outcomes in patients harboring t(11;14) compared with other risk subgroups past the first-line treatment setting.

The prognostic implications of t(11;14) in MM are important to inform new therapeutic options and the development of biomarker-targeted therapies. Since myeloma cells overexpress anti-apoptotic proteins in a heterogeneous manner, a subset of myeloma cells overexpress B-cell lymphoma-2 (BCL-2) and provide an attractive therapeutic target for BCL-2 inhibitors, such as venetoclax [22]. This is particularly relevant in patients harboring t(11;14), which is associated with BCL-2 overexpression, suggesting increased susceptibility to BCL-2 inhibitors [22]. In clinical trials, venetoclax has shown encouraging efficacy in patients with relapsed/refractory MM and t(11;14) [23, 24].

Here, we examined treatment patterns for MM and patient outcomes according to t(11;14) status in patients treated in the United States (US) de-identified database and compared these with other patient risk subgroups (patients with high-risk MM cytogenetic abnormalities [including those with co-occurrence of t(11;14)] and patients with standard-risk MM), across different lines of therapy (LoT).

## Subjects and methods

### Data source

This retrospective, observational cohort study used data from the Flatiron Health database, a US nationwide electronic health record (EHR)-derived, de-identified, longitudinal database, comprised predominantly of community-based practices. Data originated from ~280 US cancer clinics (~800 sites of care). De-identified patient-level information comprising both structured data (e.g., laboratory values and prescribed drugs) and unstructured data, which were curated via technology-enabled abstraction [25, 26] from physicians’ notes and other unstructured documents, such as pathology reports, were included. Institutional Review Board approval of the study protocol was obtained prior to study conduct, and included a waiver of informed consent. Data remained de-identified throughout the analyses to protect patient confidentiality; Flatiron Health, Inc. did not participate in these analyses.

### Patient selection

Patients with first-line MM treatment initiation (index date) between January 1, 2011, and January 31, 2020, were selected and followed up until February 29, 2020 (Supplementary Fig. S1). The index date was defined as the date of the first drug administration of an eligible MM therapy that was given within 60 days of the MM diagnosis, along with other eligible drugs, during a 28-day time window. Eligible patients were aged ≥18 years and treated in the Flatiron Health network, with at least two clinical encounters on different days occurring on or after January 1, 2011. Patients were diagnosed with MM (International Classification of Diseases, Ninth Revision [ICD-9] code 203.0x or ICD-10 diagnosis codes C90.0x or C90) and had pathology consistent with MM, verified through chart abstraction between January 1, 2011 and January 31, 2020, and were not enrolled in a clinical trial. Exclusion criteria included a lack of relevant unstructured documents, such as physicians’ notes, to verify MM diagnosis for review by the data abstraction team, patients who had been treated in the Flatiron Health network for fewer than three consecutive months, and those with Line 0 therapy, i.e., those who received MM treatment (captured through unstructured data) >30 days before the start of structured activity within the Flatiron Health network, for whom therapy data may be missing. To select patients who received recognized MM regimens of interest, patients whose first-line regimen was not recorded or not recommended by the National Comprehensive Cancer Network® (NCCN®) for the treatment of MM (monotherapy) and those receiving first-line regimens for another malignancy or initiated >60 days after the MM diagnosis date were also excluded.

### Patient cohorts and outcomes

Cytogenetic results by fluorescence in situ hybridization (FISH) were used to stratify patients into three risk cohorts: the t(11;14)+ cohort, which comprised patients with t(11;14), but excluded those with co-occurrence of high-risk cytogenetics; the high-risk cohort, which included patients with high-risk genetic abnormalities per Stratification for Myeloma and Risk-Adapted Therapy (mSMART, v3) criteria (t[4;14], t[14;16], t[14;20], chromosome 17p deletion [del(17p)], *TP53* mutation, and 1q gain) [27]; and the standard-risk cohort. Patients with t(11;14) and co-occurring high-risk cytogenetic factors were classified as high risk in this analysis.

Outcome measures included first-line, second line, and third-line treatment patterns; and time to next treatment (TTNT), defined as the time from treatment initiation to the day before the start of next treatment or death before February 29, 2020, and OS, defined as the time from treatment initiation to death.

### Sensitivity analyses

Several sensitivity analyses were conducted. Treatment patterns and outcomes according to age (<70 vs ≥70 years), as a proxy for transplant eligibility, were evaluated. OS was assessed in patients with t(11;14) with or without high-risk factors. In addition, treatment patterns and outcomes were examined in an expanded cohort of patients receiving first-line treatment, where the exclusion criterion of “non-NCCN recommended” first-line MM regimen was not applied (i.e., the “expanded first-line cohort”), thus allowing more heterogeneity in first-line treatment (patients who received doublets and monotherapies of any MM-indicated therapy were included). Finally, patient characteristics and outcomes were evaluated in the subset of patients who received the first-line triplet regimen of bortezomib, lenalidomide, and dexamethasone (VRd), the well-established and preferred initial therapy for first-line MM.

### Statistical analysis

Descriptive analyses of patient characteristics were conducted. Sankey plots were generated to describe treatment sequences by each LoT, including differentiation of the treatment and maintenance blocks. Kaplan–Meier analyses were used to estimate OS from treatment initiation until death (date of death provided at “month–year” level to protect patient privacy) and TTNT (time from treatment initiation to the day before the start of next treatment or death), by treatment regimen and MM risk subgroup. Median OS and TTNT, with corresponding 95% confidence intervals (CIs), were calculated for each risk subgroup. All patients without an event (death or next treatment) were censored at the date of last visit or last treatment administration (whichever came last) on or before February 29, 2020.

## Results

### Patient demographics and clinical characteristics

**Fig. 1:**
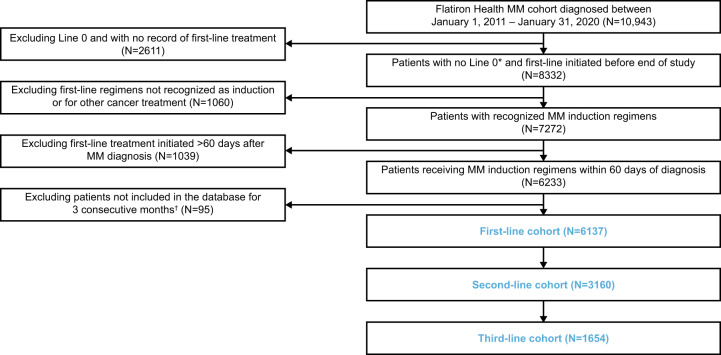
Patient disposition. *Patients who received MM treatment (as captured through unstructured data) >30 days before the start of structured activity within the Flatiron Health network, for whom therapy data may be missing. ^†^To ensure that all included patients have been in the database a sufficient duration of time to account for lags in the abstraction of data elements or data linkages to external data sources. L0 line 0, MM multiple myeloma.

A total of 10,943 patients were included in the MM cohort from January 1, 2011, through January 31, 2020, of whom 6138 met the eligibility criteria for inclusion (Fig. 1). Among these patients, 6137 received first-line treatment, 3160 received second-line treatment, and 1654 received third-line treatment. At second-line and third-line, most of the patients received NCCN-recommended regimens, while all patients involved in clinical trials were excluded (*n* = 45 at second-line, *n* = 35 at third-line); details regarding the selection based on NCCN-recommended regimens are shown in the Supplementary Methods.

Baseline characteristics per line of treatment are shown in Table 1. Overall, the median age at diagnosis was 69 years, 63% of patients were White, 16% were African American, and 54% were male. Most of the patient records originated from community practices (88%). Median follow-up times and the number of patients receiving maintenance therapy decreased as patients advanced to later lines of treatment (Table 1).

**Table 1:** Patient baseline demographics and characteristics.

	First line (*n* = 6137)	Second line (*n* = 3160)	Third line (*n* = 1654)
Sex, *n* (%)			
Female	2752 (44.8)	1438 (45.5)	769 (46.5)
Male	3385 (55.2)	1722 (54.5)	885 (53.5)
Race, *n* (%)			
White	3804 (62.0)	2018 (63.9)	1069 (64.6)
African American	982 (16.0)	518 (16.4)	276 (16.7)
Asian	110 (1.8)	60 (1.9)	27 (1.6)
Hispanic/Latino	60 (1.0)	36 (1.1)	16 (1.0)
Other	721 (11.7)	350 (11.1)	182 (11.0)
Missing	460 (7.5)	178 (5.6)	84 (5.1)
Median age at diagnosis, years [Q1, Q3]	69.0 [61.0, 76.0]	68.0 [60.0, 76.0]	67.0 [59.0, 74.0]
Practice type, *n* (%)			
Academic	680 (11.1)	395 (12.5)	223 (13.5)
Community	5457 (88.9)	2765 (87.5)	1431 (86.5)
Median follow-up time, months [Q1, Q3]	22.3 [8.85, 43.8]	18.2 [6.91, 35.9]	14.7 [5.67, 30.3]
ECOG PS, *n* (%)			
0	1222 (19.9)	692 (21.9)	379 (22.9)
1	1248 (20.3)	974 (30.8)	578 (34.9)
2	485 (7.9)	369 (11.7)	226 (13.7)
3+	168 (2.7)	95 (3.0)	51 (3.1)
Missing	3014 (49.1)	1030 (32.6)	420 (25.4)
ISS stage at diagnosis, *n* (%)			
Stage I	1152 (18.8)	578 (18.3)	302 (18.3)
Stage II	1208 (19.7)	638 (20.2)	342 (20.7)
Stage III	1254 (20.4)	686 (21.7)	368 (22.2)
Unknown/not documented	2523 (41.1)	1258 (39.8)	642 (38.8)
Prior malignancy^a^, *n* (%)			
Yes	594 (9.7)	310 (9.8)	159 (9.6)
No	5543 (90.3)	2850 (90.2)	1495 (90.4)
Received maintenance^b^ therapy, *n* (%)			
No	4854 (79.1)	2836 (89.7)	1569 (94.9)
Yes	777 (12.7)	155 (4.9)	21 (1.3)
Missing	506 (8.2)	169 (5.3)	64 (3.9)
Cytogenetics test, *n* (%)			
Results unknown/not documented	36 (0.6)	16 (0.5)	10 (0.6)
Yes	4614 (75.2)	2642 (83.6)	1422 (86.0)
No	1487 (24.2)	502 (15.9)	222 (13.4)
Cytogenetic test type, *n* (%)			
Both FISH & karyotype	3295 (53.7)	1998 (63.2)	1135 (68.6)
FISH only	829 (13.5)	375 (11.9)	161 (9.7)
Karyotype only	526 (8.6)	285 (9.0)	136 (8.2)
Missing	1487 (24.2)	502 (15.9)	222 (13.4)
FISH cytogenetic abnormality, *n* (%)			
No abnormalities identified	1478 (24.1)	878 (27.8)	453 (27.4)
Present	3172 (51.7)	1780 (56.3)	979 (59.2)
Missing	1487 (24.2)	502 (15.9)	222 (13.4)

*ECOG* *PS* Eastern Cooperative Oncology Group performance status, *FISH* fluorescence in situ hybridization, *ISS* International Staging System, *Q* quartile.

^a^Any patient with ≥1 prior malignancy.

^b^Maintenance defined only in patients who had transplants.

### Risk stratification

Altogether, 75% (*n* = 4614/6137) of patients had available FISH and karyotype test results at first-line treatment start (Supplementary Fig. S2). Of the remaining patients (*n* = 1523), 1487 had no testing, and 36 had undocumented risk with FISH (but no karyotyping); these patients were not investigated further within this analysis. The proportion of patients with FISH and karyotype results recorded was higher in later LoT (2642/3160 [84%] patients in the second-line cohort, and 1422/1654 [86%] patients in the third-line cohort, Supplementary Fig. S2). In the first-line cohort, most of the patients were grouped as standard risk (*n* = 2544; 55%), followed by high risk (1624; 35%) and t(11;14)+ (446; 10%; Supplementary Table S1). Of the 1624 patients classified as high risk in the first line, 199 (14%) also harbored t(11:14). Baseline demographics and characteristics were consistent by risk status and LoT and are presented in Supplementary Table S2.

### Treatment patterns

**Fig. 2:**
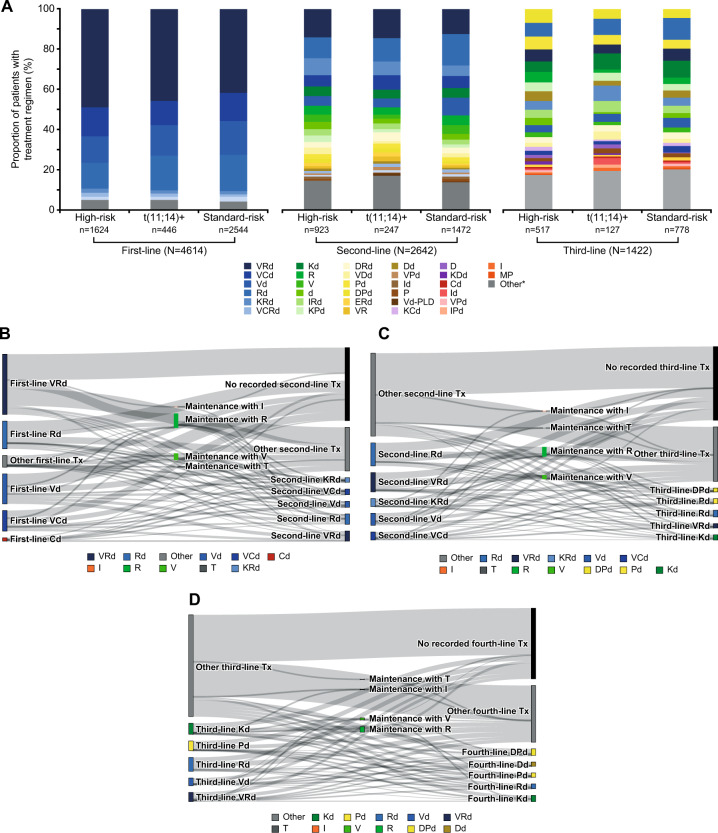
Treatment patterns per line of treatment. Proportion of patients with treatment regimen by risk subgroup (**A**), and Sankey plots showing treatment patterns for first-line (**B**), second-line (**C**), and third-line (**D**) treatment. An asterisk symbol represents all other therapies, where < 1% of patients receive a particular treatment regimen. The overall number across each line of therapy includes those patients with undocumented risk who were not included in the analyses. Therefore, the *N* indicated here is the sum of the *t*(11;14)+, high-risk and standard-risk subgroups, so does not equal the total number of patients. C cyclophosphamide, d dexamethasone, D daratumumab, E elotuzumab, I ixazomib, K carfilzomib, M melphalan, P pomalidomide, PLD pegylated liposomal doxorubicin, R lenalidomide, t translocation, V bortezomib.

VRd was the most common first-line treatment (received by >40% of patients) across all cytogenetic risk subgroups studied (Fig. 2A, B). VRd and Rd were the two most common second-line combination treatments across the cytogenetic risk subgroups, with carfilzomib emerging as a second-line option (in combination with Rd for ~5% of patients, dexamethasone for ~7% of patients, and pomalidomide plus dexamethasone for ~4% of patients; Fig. 2A, C). The third-line treatment pattern was fragmented, with numerous different treatment regimens utilized (Fig. 2A, D). The use of daratumumab and elotuzumab was more frequent in the third line compared with earlier lines of therapy. The use of triplet therapy decreased as patients advanced to later LoT (46% for first-line, 30% for second-line, and 26% for third-line therapy), whereas the use of doublets increased (28% for first-line, 30% for second-line, and 34% for third-line therapy).

Across all treatment lines, the most common MM treatment regimens captured in the database were PI plus steroid plus IMiD, steroid plus IMiD, PI plus steroid, and PI plus chemotherapy plus steroid (Supplementary Table S3). In each therapy line, patients with different cytogenetic statuses received similar treatment: in the first-line, ~85% of patients in the high-risk subgroup received PI-containing regimens, compared with ~79% and ~78% for the t(11;14)+ and standard-risk subgroups, respectively. In the second line, ~63% of patients in the high-risk subgroup, ~62% in the t(11;14)+ subgroup, and ~59% in the standard-risk subgroup received PI-containing regimens, whereas in the third-line, receipt of PI-containing regimens was slightly lower in the high-risk subgroup than in the t(11;14)+ and standard-risk subgroups (~49% vs ~58% and ~52%, respectively).

### Clinical outcomes (TTNT and OS)

**Fig. 3:**
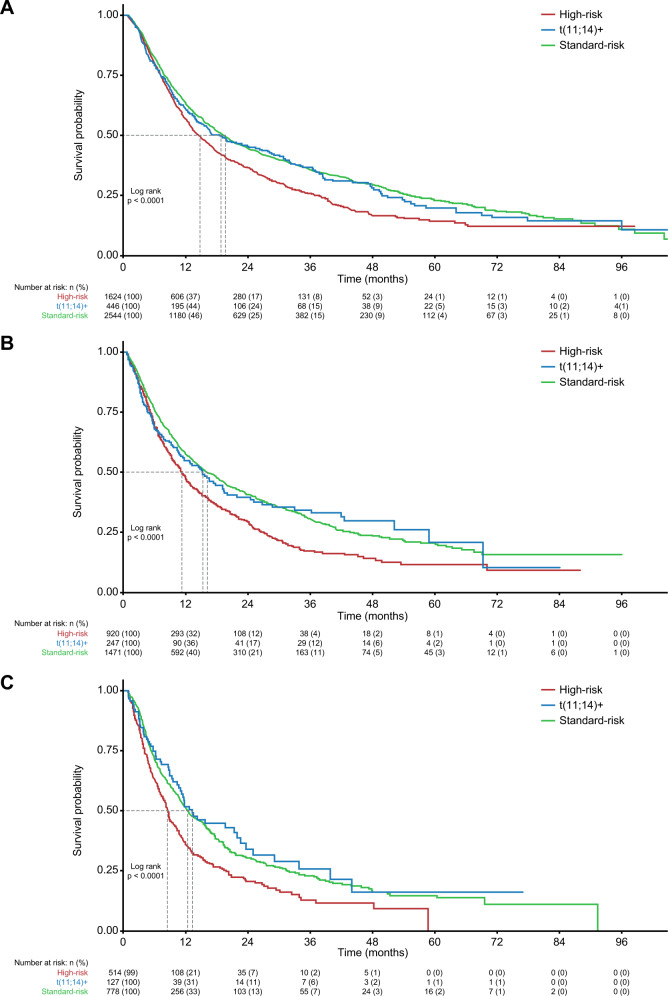
Kaplan–Meier curves for TTNT by patient risk subgroup. First-line (**A**), second-line (**B**), and third-line (**C**) therapy. t translocation, TTNT time to next treatment.

TTNT decreased as patients advanced to later LoT (Fig. 3); median TTNT (95% CI) was shorter for patients in the high-risk subgroup than for those in the t(11;14)+ and standard-risk subgroups across all LoT. In the first-line setting, median (95% CI) TTNT was 14.8 (13.4–16.3), 18.8 (15.2–26.7), and 19.6 (18.0–21.2), months for the high-risk, t(11;14)+, and standard-risk groups, respectively. Corresponding median (95% CI) TTNT durations in second-line were 11.5 (10.3–12.7), 15.5 (11.7–19.7), and 16.5 (14.7–18.8) months, respectively, and in third-line were 8.6 (7.8–9.6), 13.3 (11.1–22.6), and 12.4 (11.2–15.0) months, respectively.

**Fig. 4:**
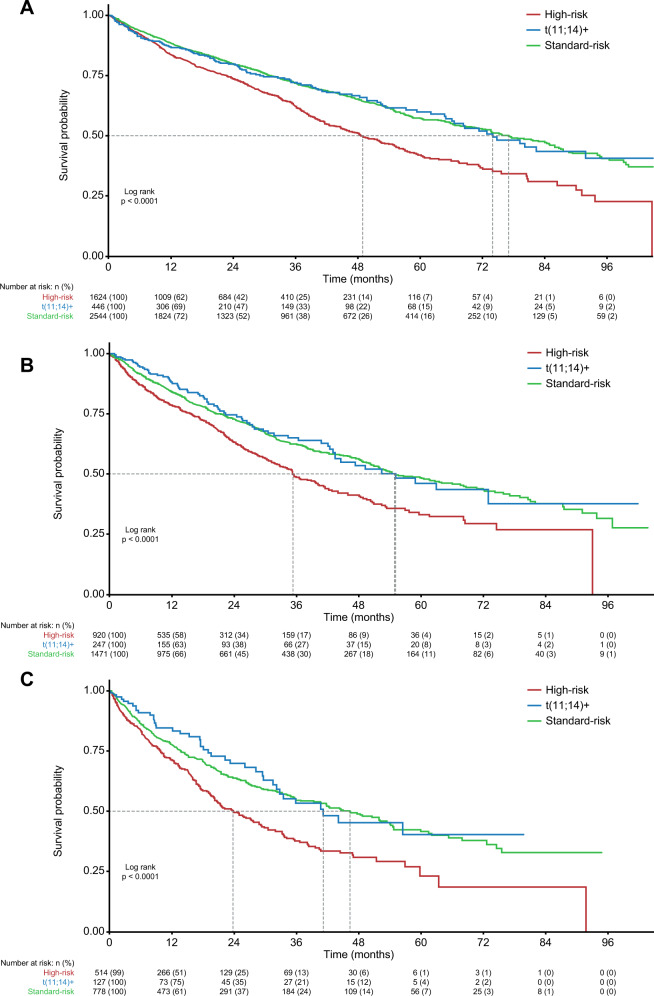
Kaplan–Meier curves for OS by patient risk subgroup. First-line (**A**), second-line (**B**), and third-line (**C**) treatment. OS overall survival, t translocation.

Across all LoT, patients in the high-risk subgroup had the poorest median OS compared with patients in the t(11;14)+ and standard-risk subgroups (Fig. 4). In the first-line setting, median (95% CI) OS was 48.9 (45.0–55.4), 74.0 (66.1–not reached [NR]), and 77.0 (71.9–84.9), months, in the high-risk, t(11;14)+, and standard-risk groups, respectively, while corresponding median OS values in second-line and third-line were 35.3 (32.2–40.4), 55.1 (43.4–NR), and 54.8 (50.9–67.5) months, respectively, and 23.8 (20.3–29.1), 41.1 (32.1–NR), and 46.3 (39.3–54.1) months, respectively.

### Sensitivity analyses

#### First-line treatment patterns and outcomes in patients aged <70 versus ≥70 years

VRd was the most common first-line treatment regimen both for patients aged <70 years and those aged ≥70 years (1525 [49%] and 1095 [36%] patients, respectively). Further, more patients aged ≥70 years were treated with doublet therapy Rd or bortezomib plus dexamethasone (Vd) than those aged <70 years (Supplementary Fig. S3A).

TTNT across the three cytogenetic risk subgroups did not seem to be affected by patient age; stratifying based on age <70 versus ≥70 years did not show any trends (Supplementary Fig. S3B). In contrast, a trend toward inferior OS for patients aged ≥70 years was evident across the different risk subgroups (Supplementary Fig. S3C). In addition, OS for patients aged <70 years in the high-risk subgroup was similar to that in patients aged ≥70 years in the t(11;14)+ and standard-risk subgroups.

#### OS in patients with t(11;14) with or without high-risk factors

Median OS was longest in patients with t(11;14) and no high-risk factors (74 months [95% CI 66.1–NR]) and shortest in patients with t(11;14) and any high-risk factors (41.9 months [37.5–NR]; Supplementary Fig. S4 and Supplementary Table S4).

#### Patient characteristics, treatment patterns, and outcomes for the overall expanded first-line cohort

Among the 6944 patients included in the expanded first-line cohort, 5140 (74%) had FISH and karyotype test results before the initiation of first-line treatment, with 491 (10%) categorized as t(11;14)+, 1786 (35%) as high risk, and 2863 (56%) as a standard risk; 158 unique treatment combinations were utilized. The most common treatment regimens were VRd (2636 [38%] patients), Rd (1082 [16%]), Vd (1002 [14%]), bortezomib plus cyclophosphamide and dexamethasone (818 [12%]), and dexamethasone monotherapy (323 [5%]) (Supplementary Fig. S5A).

Within the expanded first-line cohort, patients in the high-risk subgroup had the shortest median TTNT (95% CI) of 13.9 (13.0–15.4) months compared with 16.9 (14.2–24.2) months in the t(11;14)+ subgroup and 18.8 (17.4–20.3) months in the standard-risk subgroup (Supplementary Fig. S5B). Patients in the high-risk subgroup also had the shortest median OS (95% CI): 48.5 (44.1–53.5) months compared with 74.0 (64.9–NR) months and 73.7 (67.8–78.8) months in the t(11;14)+ and standard-risk subgroups, respectively (Supplementary Fig. S5C).

#### Patient characteristics and outcomes in patients receiving first-line VRd

**Fig. 5:**
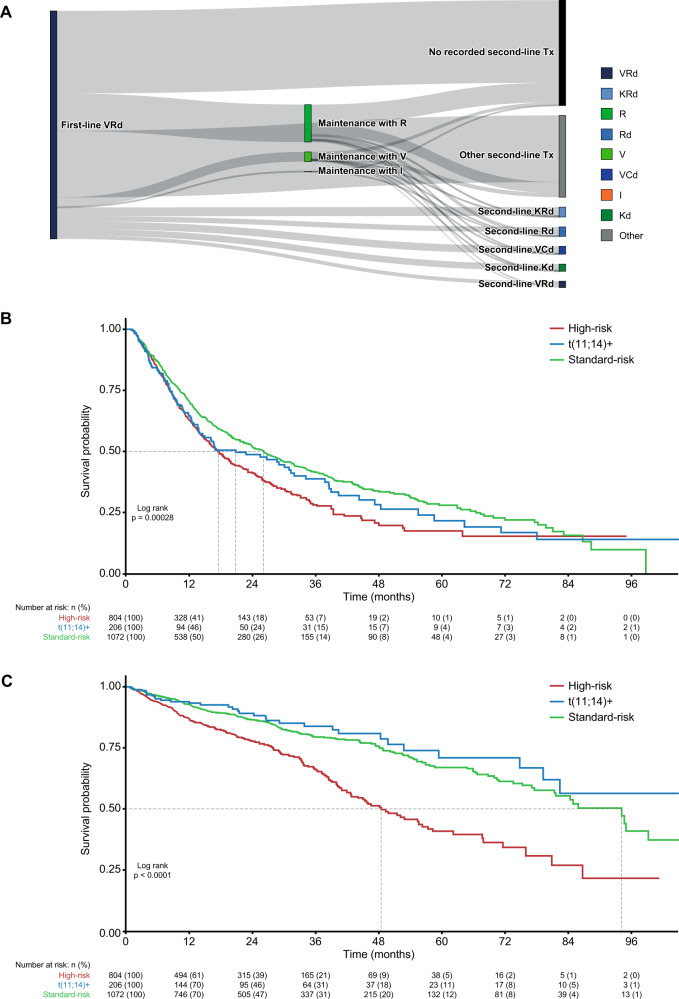
Sensitivity analysis in patients who received first-line VRd. Sankey plot showing treatment patterns (**A**), and Kaplan–Meier curves for TTNT (**B**) and OS (**C**). OS overall survival, t translocation, TTNT time to next treatment, VRd bortezomib lenalidomide dexamethasone.

The overall treatment pattern for patients who received the VRd regimen (*n* = 2636) is presented in Fig. 5A. Of these patients, 2082 (79%) had FISH and karyotype test results before the start of therapy, with 804 (39%) categorized as high-risk, 206 (10%) as t(11;14)+, and 1072 (51%) as standard-risk (Supplementary Table S5).

Median TTNT (95% CI) for patients in the high-risk, t(11;14)+, and standard-risk subgroups was 17.5 (15.7–19.9), 20.8 (14.5–31.9), and 26.1 (22.8–29.8) months, respectively (Fig. 5B). Median OS (95% CI) for patients in the high-risk, t(11;14)+, and standard-risk subgroups was 48.4 (42.8–57.4), not estimable (79.2–NR), and 94.1 (81.6–NR) months, respectively (Fig. 5C).

## Discussion

In a predominantly community-based setting in the US, from January 2011 to the end of February 2020, similar treatment patterns were observed for patients with t(11;14) compared with patients categorized as high and standard risk in the first-line and second-line settings. Across all LoT, the outcomes of patients in the high-risk subgroup were less favorable than those for patients in the t(11;14)+ and standard-risk subgroups. Although, of note, almost one-third (199/645, 31%) of patients with t(11;14) were allocated to the high-risk subgroup for analysis due to co-occurrence of high-risk genetic factors. As t(11;14) is an early genomic event, it is possible that increased genomic instability could occur with later LoT [28]. Thus, the presence of t(11;14) could be considered as an intermediate risk factor, particularly when additional high-risk genetic aberrations co-occur as the disease progresses. This presents the opportunity for future investigations to assess whether earlier interventions with targeted treatment achieve superior outcomes in patients with t(11;14).

Overall, the prevalence of t(11;14) in this analysis (14% for patients in the first-line setting with results recorded) was similar to the general prevalence reported in ECOG trials and the IFM clinical trials (~16%) [15, 29]. In addition, patient demographics in this study are comparable with those reported for other US real-world studies, including International Oncology Network (ION) electronic medical record data [30], Surveillance, Epidemiology and End Results (SEER) Medicare data [31], and the Optum database [32]. Median age at diagnosis in the current study was 69 years compared with 69 and 71 years in the ION [30] and Optum databases [32], respectively. A higher representation of non-White patients was included in this study: 64% of patients in the current study were White versus 52% from the ION report [30]. In addition, ~16% of patients were African American versus 18% in the SEER Medicare database [33]. Furthermore, in the current study, patients were treated primarily in the community setting (88%), which largely reflects general patterns of care in the US [34]. The transplant rate in first-line was lower in this study cohort than other US cohorts (26% of patients, compared with 37% in the CONNECT MM registry) [35].

Most of the first-line and second-line treatment regimens included bortezomib and/or lenalidomide (VRd was the most common treatment regimen for first-line therapy), consistent with other real-world data studies in the US [30]. Substantial variation was observed in third-line treatments, with no single treatment or regimen dominating the landscape, although more recently approved agents, such as daratumumab became more prominent, also mirroring other reports on MM treatment [30]. Although overall triplet therapy in first-line was lower than may be expected, this is a result of the time period over which this study was conducted, where doublet therapy was more common in the earlier years and triplet therapy more common in later years [37]. It is also important to note that, by the end of 2014, various approvals had been made for treatment regimens for relapsed/refractory MM, thus shifting the treatment paradigm. As the present study includes patients treated between January 1, 2011 and February 29, 2020, all treatments during this time are considered equally; thus, any treatment paradigm shifts that occurred due to new approvals of treatment regimens or updates to the initially approved indications (e.g., treatments used in 2011 may have been replaced by newer regimens) are not specifically addressed in this analysis but have been reported previously [36, 37].

Median TTNT decreased in later treatment lines across all risk subgroups, suggesting that treatment benefit decreases over time, perhaps in part related to disease progression or development of resistance [38, 39]. High-risk patients consistently had poorer clinical outcomes (TTNT and OS) compared with the t(11;14)+ and standard-risk subgroups, irrespective of therapy line, similar to findings reported previously [40, 41]. Moreover, patients with t(11;14) and high-risk factors had inferior survival outcomes than those with t(11;14) alone.

In the current analysis, median TTNT and OS in the t(11;14)+ group were similar to those observed for the standard-risk group. In contrast, Bal et al. found that trends for OS and progression-free survival (PFS) following first-line treatment were worse in patients with t(11;14) compared with those without t(11;14) [21]. In addition, they found that outcomes for patients with t(11;14) were worse than in those without t(11;14) when evaluating patients treated with first-line PIs. However, no difference was seen in patients who received IMiD or PI plus IMiD-based therapy, or autologous hematopoietic cell transplantation in the first-line setting [21]. Although both studies use the Flatiron Health database, it should be noted that there are several differences between the analysis in this study and that by Bal et al. “High risk” was defined in the current study using the mSMART (v3) criteria and included patients with co-occurrence of t(11;14)+, while in the Bal et al. study, several high-risk cohorts such as patients with del(17p), chromosome 1 abnormalities, and high-risk translocations were defined [21]. Further, real-world derived PFS (rwPFS) was used as an endpoint in the Bal et al. analysis, which is derived from results that were only available for ~50% of the MM patients in the Flatiron Health database. Missing data could lead to a potential overestimation of rwPFS and may or may not be a differential between the studied cohorts [21]. Therefore, we used TTNT as a proxy of progression, as it provides information on patients for whom progression status is unknown.

A trend toward inferior OS was observed for patients aged ≥70 years across the different risk subgroups. Inferior OS is well documented among this patient group [42], and may be due to the proportionately greater use of doublet (i.e., Rd and Vd) versus triplet therapies that was observed in patients aged ≥70 years in this study. Rd and Vd have been identified as regimens in patients with transplant-ineligible MM [43, 44], as elderly patients may not be able to tolerate triplet therapies as well as younger patients [43].

Among the first-line VRd-treated population, patients in the t(11;14)+ and standard-risk subgroups had similar OS, which was longer than that reported for the high-risk subgroup. TTNT for the t(11;14)+ subgroup was shorter than TTNT for the standard-risk subgroup (but longer than the high-risk subgroup), indicating that VRd has less impact on patients deemed high-risk, and potentially those with t(11;14). Thus, these data suggest a need for exploration of other treatment methods, such as targeted therapies. In the first-line expanded cohort, which had increased heterogeneity in the treatment regimens used, all risk subgroups had numerically shorter TTNT compared with the first-line cohort from the main analysis (where the exclusion criterion of “non-NCCN recommended” first-line MM regimen was applied). Median OS was similar in the t(11;14)+ and high-risk subgroups of the expanded first-line study compared with the first-line cohort from the main analysis, and shorter in patients in the standard-risk subgroup (74 vs 77 months overall).

New treatment regimens are currently under investigation, so it is likely that the treatment landscape of MM will continue to develop, with the continued evolution of treatment sequencing in MM. Several novel drug classes have recently been approved by the US Food and Drug Administration for the treatment of relapsed/refractory MM, including the anti-B-cell maturation antigen chimeric antigen receptor T-cell therapy, idecabtagene vicleucel (ide-cel) [45], selinexor, in combination with Vd [46], and belantamab mafodotin–blmf [47]. The BCL-2 inhibitor venetoclax is under investigation for relapsed/refractory MM, specifically to assess effectiveness in patients carrying t(11;14) following the phase 3 BELLINI trial (NCT02755597) of venetoclax plus Vd versus placebo plus Vd, that demonstrated an increased risk of mortality in the overall population but a more favorable risk–benefit profile in patients with t(11;14) or BCL-2-high gene expression [48]. Venetoclax continues to be explored in combination with other agents; noteworthy are the ongoing phase 3 CANOVA trial (NCT03539744) of venetoclax plus dexamethasone versus pomalidomide plus dexamethasone [49], the phase 1 trial (NCT03314181) of venetoclax plus daratumumab and dexamethasone with and without bortezomib [50], and the phase 1/2 trial (NCT01794520) of venetoclax monotherapy and venetoclax plus dexamethasone therapy [51, 52] in patients with t(11;14)+ relapsed/refractory MM.

Since Flatiron Health data are obtained primarily (>80% of data) from provider EHRs for patients at participating community centers, caution should be taken when generalizing these findings to a broader patient population—in particular, the lower proportion of patients in this cohort with a transplant [35]. We did not have full medical history at baseline to determine factors related to transplant eligibility at diagnosis, thus we used age ≥70 years as a proxy. Another potential limitation of the current study is that next-generation sequencing testing information, permitting further characterization of high-risk patients, was not available in the Flatiron Health MM database.

## Conclusions

This analysis of predominantly community-based treatment patterns, spanning from 2011 to 2020, suggests that in the absence of biomarker-driven therapy, treatment patterns remain similar across LoT in high-risk, t(11;14)+ and standard-risk subsets of patients. As such, novel therapeutics targeted to defined subgroups offer an opportunity for more personalized medicine in MM based on genomic profiles to optimize patient outcomes. This is true not just in high-risk populations, but also in other defined subgroups such as patients with t(11;14)+ MM, a population for whom one-third also harbor additional high-risk cytogenetic features.

## Supplementary information

Supplementary Material
