# Supplementary material for: Treatment patterns and outcomes according to cytogenetic risk stratification in patients with multiple myeloma: a real-world analysis
Source: Blood Cancer J. 2022 Mar 23;12(3):46. doi: 10.1038/s41408-022-00638-0 (PMC8943165; doi:10.1038/s41408-022-00638-0)
Supplement: Supplementary file 1 — Supplementary Material [file 41408_2022_638_MOESM1_ESM.pdf]

SUPPLEMENTARY METHODS

Classification of therapy and eligibility criteria based on treatment

Line of therapy (LoT) was derived from a complex set of data, incorporating structured data elements from therapies ordered and administered and abstracted data elements from transplant and oral therapies (start date and stop date).

In brief, a LoT was generally the first eligible drug episode plus other eligible drugs given within 28 days. The start of the first LoT (LoT1) was the date of the first drug episode observed as structured data in the network; that is, an eligible therapy initiated within the window of 14 days before the diagnosis date or after the diagnosis date, but after the start of structured activity (earliest structured visit or therapy). The name of the "regimen" for that LoT is the combination of therapies in that line, (within 28 days) unless otherwise noted.

A regimen such as induction of bortezomib, lenalidomide, and dexamethasone (VRd) may form one block of a LoT; if a patient also received a transplant, that would form an additional block of a LoT (starting on the date on which the transplanted cells are infused). If a patient then subsequently received a maintenance regimen (exceeding 60 days of continuous therapy, and following transplant by less than 6 months), that was also considered a block within the same LoT. Maintenance regimens following an induction combination (without transplant) were a separate block within the LoT. Transplant and maintenance regimens following these rules did not advance the LoT.

Eligibility criteria

Following evaluation of the treatment in the Flatiron Health database, the study team considered the following eligibility criteria to be applicable when conducting studies to understand treatment patterns or treatment of patients with multiple myeloma (MM):

- Exclude patients with no treatment received or documented
- Exclude "Line 0" patients: "Line 0" is a flag created by Flatiron Health to indicate patients for whom early treatment data may be missing from the database. These patients have some evidence (through unstructured data) of MM treatment occurring >30 days before the start of structured activity in the Flatiron Health network. In these patients, Line 0 extends from the start of MM treatment to the start of structured activity, but does not contain complete information on specific therapies. Therefore, assumptions about what LoT is labeled as LoT1 may be misclassified if these patients are included
- Exclude patients with a record of "Clinical Study Drug" in the LoT of interest (patients would not be excluded from a first-line cohort if they had a clinical study drug in LoT3, but for analyses of second-line or third-line cohorts, patients receiving clinical study drug in the line of interest are excluded)
- Exclude patients with combination regimens indicated for the active treatment of cancers other than MM in LoT1 e.g., cisplatin plus etoposide; (or any other LoT where a study is indexed on that LoT); however, common maintenance therapies for other cancers could be co-administered with MM therapies, and there is not a strong enough rationale to exclude patients who were co-administered maintenance therapies for other cancers. These co-administered therapies included anastrozole, leuprolide, hydroxyurea, letrozole,

methotrexate, tamoxifen, bicalutamide, abiraterone, fulvestrant, exemestane, and capecitabine. Where these occurred in a LoT, they were either:
  - Removed from the LoT label, or
  - Ignored where they were stand-alone therapies in a LoT, or
  - Suppressed from advancing the LoT.
- Exclude patients who were not receiving recognized or indicated MM induction regimens in first-line according to National Comprehensive Cancer Network (NCCN) guidelines, and/or non-indicated first-line combinations e.g., a pomalidomide-containing regimen in first-line or monotherapy in first-line
  - A total of 47 combinations of MM therapies were considered

Certain additional modifications were made:
- In first-line, we observed a proportion of patients for whom an immunomodulating drug or proteasome inhibitor or steroid was observed administered as monotherapy and then a combination regimen was initiated >28 days after the start of the monotherapy, which according to the LoT rules would be a second-line. For example, bortezomib being initiated as 1–2 cycles of monotherapy (>28 days) before cyclophosphamide plus dexamethasone or lenalidomide plus dexamethasone are added to complete the induction regimen (CVd or VRd, respectively). Where this gap was less than 60 days, we merged and considered this a first-line combination
- Modified classification of maintenance post-transplant: maintenance therapies as recognized by the NCCN are lenalidomide, bortezomib, and ixazomib as monotherapy, and combination bortezomib plus lenalidomide. There were ~280 patients where these regimens appeared in a stand-alone LoT directly following a transplant (from a previous LoT) but were not classified as maintenance in Flatiron rules. Based on minimal evidence of progression in the response data (<10 cases) at the end of the transplant block and preceding the LoT containing the maintenance regimen, these regimens were reclassified as maintenance and rolled into the prior LoT, containing maintenance as a treatment block within the LoT

SUPPLEMENTARY RESULTS

Table S1 Cytogenetics (FISH and karyotype) classification.

Cytogenetic classification, n (%)	First-line (n=4614)	Second-line (n=2642)	Third-line (n=1422)
All patients with t(11;14), regardless of other high-risk factors	645 (14.0)	372 (14.1)	201 (14.1)
**t(11;14) without high-risk factors**	**446 (9.7)**	**247 (9.3)**	**127 (8.9)**
t(11;14) with any high-risk factor	199 (4.3)	125 (4.7)	74 (5.2)
t(11;14) with del(17p)	76 (1.6)	46 (1.7)	29 (2.0)
**High-risk***	**1624 (35.2)**	**923 (34.9)**	**517 (36.4)**
High-risk with t(4;14), t(14;16), or del(17p)	755 (16.4)	447 (16.9)	261 (18.4)
**Standard-risk**	**2544 (55.1)**	**1472 (55.7)**	**778 (54.7)**

The three subgroups considered in the current analysis are highlighted in bold.

*Includes all patients with high-risk factors (including chromosome 1 abnormalities), and those with co-occurrence of t(11;14).

del deletion, *t* translocation.

Table S2 Baseline demographics and characteristics by risk status.

	First-line (n=6137)				Second-line (n=3160)				Third-line (n=1654)			
	HR (n=1624)	t(11;14)+ (n=446)	SR (n=2544)	UR (n=1523)	HR (n=923)	t(11;14)+ (n=247)	SR (n=1472)	UR (n=518)	HR (n=517)	t(11;14)+ (n=127)	SR (n=778)	UR (n=232)
Sex, n (%)												
Male	837 (52)	270 (61)	1452 (57)	826 (54)	473 (51)	151 (61)	846 (58)	252 (49)	271 (52)	73 (58)	436 (56)	105 (45)
Race, n (%)												
White	990 (61)	279 (63)	1,595 (63)	940 (62)	598 (65)	160 (65)	922 (63)	338 (65)	337 (65)	79 (62)	499 (64)	154 (66)
African American	240 (15)	70 (16)	434 (17)	238 (16)	128 (14)	36 (15)	272 (19)	82 (16)	82 (16)	18 (14)	138 (18)	38 (16)
Asian	37 (2)	5 (1)	49 (2)	19 (1)	23 (3)	1 (<1)	30 (2)	6 (1)	10 (2)	1 (1)	16 (2)	0
Other	207 (13)	53 (12)	317 (12)	204 (13)	113 (12)	30 (12)	179 (12)	64 (12)	60 (12)	20 (16)	88 (11)	30 (13)
Missing	150 (9)	39 (9)	149 (6)	122 (8)	61 (7)	20 (8)	69 (5)	28 (5)	28 (5)	9 (7)	37 (5)	10 (4)
Median age at diagnosis, years [Q1, Q3]	69 [62, 76]	69 [61, 76]	69 [60, 76]	70 [62, 77]	68 [60, 75]	68 [61, 77]	67 [59, 75]	70 [62, 77]	67 [59, 74]	66 [60, 74]	66 [58, 74]	70 [63, 76]
Practice type, n (%)												
Academic	158 (10)	46 (10)	234 (9)	242 (16)	112 (12)	30 (12)	162 (11)	91 (18)	69 (13)	17 (13)	95 (12)	42 (18)
Community	1466 (90)	400 (90)	2310 (91)	1281 (84)	811 (88)	217 (88)	1310 (89)	427 (82)	448 (87)	110 (87)	683 (88)	190 (82)
Median follow-up time, months [Q1, Q3]	19 [8, 36]	22 [10, 45]	26 [11, 49]	21 [8, 43]	16 [6, 31]	17 [7, 38]	20 [8, 41]	17 [6, 35]	13 [5, 24]	16 [6, 32]	17 [7, 35]	12 [4, 28]
ECOG PS, n (%)												
0	380 (23)	108 (24)	500 (20)	234 (15)	195 (21)	56 (23)	336 (23)	105 (20)	112 (22)	35 (28)	186 (24)	46 (20)
1	346 (21)	96 (22)	542 (21)	264 (17)	278 (30)	81 (33)	469 (32)	146 (28)	182 (35)	45 (35)	269 (35)	82 (35)
2	135 (8)	23 (5)	192 (8)	135 (9)	117 (13)	26 (11)	157 (11)	69 (13)	67 (13)	16 (13)	104 (13)	39 (17)
3	41 (3)	14 (3)	57 (2)	56 (4)	35 (4)	5 (2)	37 (3)	18 (4)	19 (4)	4 (3)	16 (2)	12 (5)
Missing	722 (45)	205 (46)	1253 (49)	834 (55)	298 (32)	79 (32)	473 (32)	180 (35)	137 (27)	27 (21)	203 (26)	53 (23)
ISS stage at diagnosis, n (%)												
Stage I	273 (17)	107 (24)	521 (21)	251 (17)	140 (15)	55 (22)	301 (20)	82 (16)	82 (16)	25 (20)	160 (21)	35 (15)
Stage II	355 (22)	103 (23)	501 (20)	249 (16)	203 (22)	68 (28)	274 (19)	93 (18)	119 (23)	36 (28)	144 (19)	43 (19)
Stage III	396 (24)	84 (19)	507 (20)	267 (18)	246 (27)	47 (19)	295 (20)	98 (19)	139 (27)	23 (18)	167 (22)	39 (17)
Unknown	600 (37)	152 (34)	1,015 (40)	756 (50)	334 (36)	77 (31)	602 (41)	245 (47)	177 (34)	43 (34)	307 (40)	115 (50)

	First-line (n=6137)				Second-line (n=3160)				Third-line (n=1654)			
	**HR (n=1624)**	**t(11;14)+ (n=446)**	**SR (n=2544)**	**UR (n=1523)**	**HR (n=923)**	**t(11;14)+ (n=247)**	**SR (n=1472)**	**UR (n=518)**	**HR (n=517)**	**t(11;14)+ (n=127)**	**SR (n=778)**	**UR (n=232)**
Prior malignancy, n (%)												
Yes	151 (9)	38 (9)	236 (9)	169 (11)	84 (9)	22 (9)	151 (10)	53 (10)	41 (8)	11 (9)	83 (11)	24 (10)
No	1473 (91)	408 (92)	2308 (91)	1354 (89)	839 (91)	225 (91)	1321 (90)	465 (90)	476 (92)	116 (91)	695 (89)	208 (90)
Received maintenance therapy, n (%)												
No	1249 (77)	339 (76)	1979 (78)	1287 (85)	821 (89)	228 (92)	1311 (89)	476 (92)	490 (95)	117 (92)	735 (95)	227 (98)
Yes	211 (13)	71 (16)	356 (14)	139 (9)	42 (5)	7 (3)	86 (6)	20 (4)	7 (1)	0	13 (2)	1 (<1)
Missing	164 (10)	36 (8)	209 (8)	97 (6)	60 (7)	12 (5)	75 (5)	22 (4)	20 (4)	10 (8)	30 (4)	4 (2)
Cytogenetic test type, n (%)												
FISH & karyotype	1280 (79)	350 (79)	1665 (65)	0	766 (83)	206 (83)	1026 (70)	0	452 (87)	112 (88)	571 (73)	0
FISH only	344 (21)	96 (22)	353 (14)	36 (2)	157 (17)	41 (17)	161 (11)	16 (3)	65 (13)	15 (12)	71 (9)	10 (4)
Karyotype only	0	0	526 (21)	0	0	0	285 (19)	0	0	0	136 (18)	0
Missing	0	0	0	1487 (98)	0	0	0	502 (97)	0	0	0	222 (96)
FISH cytogenetic abnormality, n (%)												
No abnormalities	0	0	1442 (57)	36 (2)	0	0	862 (59)	16 (3)	0	0	443 (57)	10 (4)
Present	1624 (100)	446 (100)	1102 (43)	0	923 (100)	247 (100)	610 (41)	0	517 (100)	127 (100)	335 (43)	0
Missing	0	0	0	1487 (98)	0	0	0	502 (97)	0	0	0	222 (96)
Median karyotype cytogenetic abnormality, no. of chromosomes [Q1, Q3]	46 [45, 53]	46 [45, 56]	51 [46, 54]	51 [47, 55]	47 [45, 53]	46 [45, 46]	52 [46, 54]	NA	47 [45, 53]	46 [45, 46]	52 [46, 55]	NA

ECOG PS Eastern Cooperative Oncology Group performance status, *FISH* fluorescence in situ hybridization, *HR* high-risk; *ISS* International Staging System, *Q* quartile, *SR* standard-risk, *t* translocation, *UR* undocumented risk.

Table S3 Treatment classes per line of treatment.

Treatment class	First-line (n=6137)	Second-line (n=3160)	Third-line (n=1654)
PI + steroid + IMiD	2779 (45.3)	835 (26.4)	340 (20.6)
Steroid + IMiD	1102 (18.0)	559 (17.7)	264 (16.0)
PI + steroid	1010 (16.5)	416 (13.2)	222 (13.4)
PI + chemotherapy + steroid	878 (14.3)	251 (7.9)	98 (5.9)
Steroid + chemotherapy	141 (2.3)	83 (2.6)	40 (2.4)
PI + steroid + IMiD + chemotherapy	115 (1.9)	64 (2.0)	24 (1.5)
Steroid + chemotherapy + IMiD	47 (0.8)	34 (1.1)	20 (1.2)
IMiD + PI	27 (0.4)	70 (2.2)	32 (1.9)
PI + mAb + IMiD + steroid	14 (0.2)	30 (1.0)	25 (1.5)
Steroid + IMiD + mAb	11 (0.2)	178 (5.6)	170 (10.3)
PI + steroid + mAb	10 (0.2)	84 (2.7)	53 (3.2)
IMiD monotherapy	–	183 (5.8)	92 (5.6)
PI monotherapy	–	133 (4.2)	66 (4.0)
Steroid monotherapy	–	100 (3.2)	44 (2.7)
Other*	–	63 (2.0)	53 (3.2)
Steroid + mAb	–	42 (1.3)	71 (4.3)
Chemotherapy alone	–	13 (0.4)	9 (0.5)
mAb monotherapy	–	11 (0.4)	20 (1.2)
mAb + IMiD	–	8 (0.3)	10 (0.6)

*Other includes MM-indicated therapies in combination with other systemic anticancer therapies.

Treatment classes used in at least 5 patients are displayed.

Chemotherapy: Cyclophosphamide, doxorubicin, melphalan; vincristine or bendamustine.
IMiD: Lenalidomide, pomalidomide or thalidomide.
mAb: Daratumumab, elotuzumab or isatuximab.
PI: Bortezomib, carfilzomib, or ixazomib.
Steroid: Dexamethasone, prednisone methylprednisolone, prednisolone.

IMiD immunomodulating drug, *mAb* monoclonal antibody, *MM* multiple myeloma, *PI* proteosome inhibitor.

Table S4 Median OS for patients with t(11;14) with or without high-risk factors.

	N	Events	Median, months	95% CI, months
t(11;14) all-comers	645	196	68.6	60.5–91.9
t(11;14) with no high-risk factors	446	127	74.0	66.1–NE
t(11;14) with any high-risk factors	199	69	41.9	37.5–NE
t(11;14) with del(17p)	76	35	33.1	12.9–NE
t(11;14) with remaining high risk*	142	41	NE	40.1–NE

*Patients with high-risk factors other than or in addition to del(17p).

CI confidence interval, *del* deletion, *NE* not estimable, *OS* overall survival, *t* translocation.

Table S5 Maintenance therapy, transplant, and cytogenetics classification (FISH and karyotyping) for the VRd sensitivity analysis cohort.

	**First-line (n=2636)**
**Received maintenance* therapy, n (%)**	
No	1889 (71.7)
Yes	500 (19.0)
Missing	247 (9.4)
**Received transplant in first-line or second-line, n (%)**	
No	1889 (71.7)
Yes	747 (28.3)
**Cytogenetics test, n (%)**	
Results unknown/not documented	540 (2.0.5)
Yes	2082 (79.0)
No	14 (0.5)
**Cytogenetic classification, n (% of patients with cytogenetic test results [n=2082])**	
All patients with t(11;14), regardless of other high-risk factors	312 (15.0)
**t(11;14) without high-risk factors**	**206 (9.9)**
t(11;14) with any high-risk factor	106 (5.1)
t(11;14) with del(17p)	42 (2.0)
**High-risk†**	**804 (38.6)**
High-risk with t(4;14), t(14;16), or del(17p)	372 (17.9)
**Standard-risk**	**1,072 (51.5)**

The three subgroups considered in the current analysis are highlighted in bold.

*Maintenance defined only in patients who had transplants.

†Includes all patients with high-risk factors (including chromosome 1 abnormalities) and those with co-occurrence of t(11;14).

FISH fluorescence in situ hybridization, *t* translocation, *VRd* bortezomib, lenalidomide, and dexamethasone.

Fig. S1 Study duration and treatment lines.

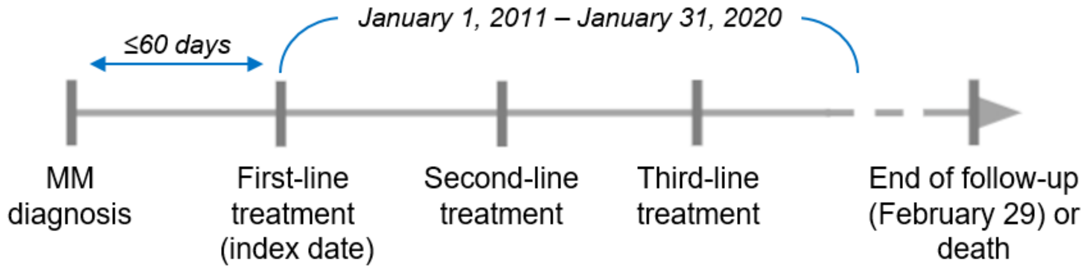

MM multiple myeloma.

Fig. S2 Availability of FISH and karyotype results before line of treatment initiation.

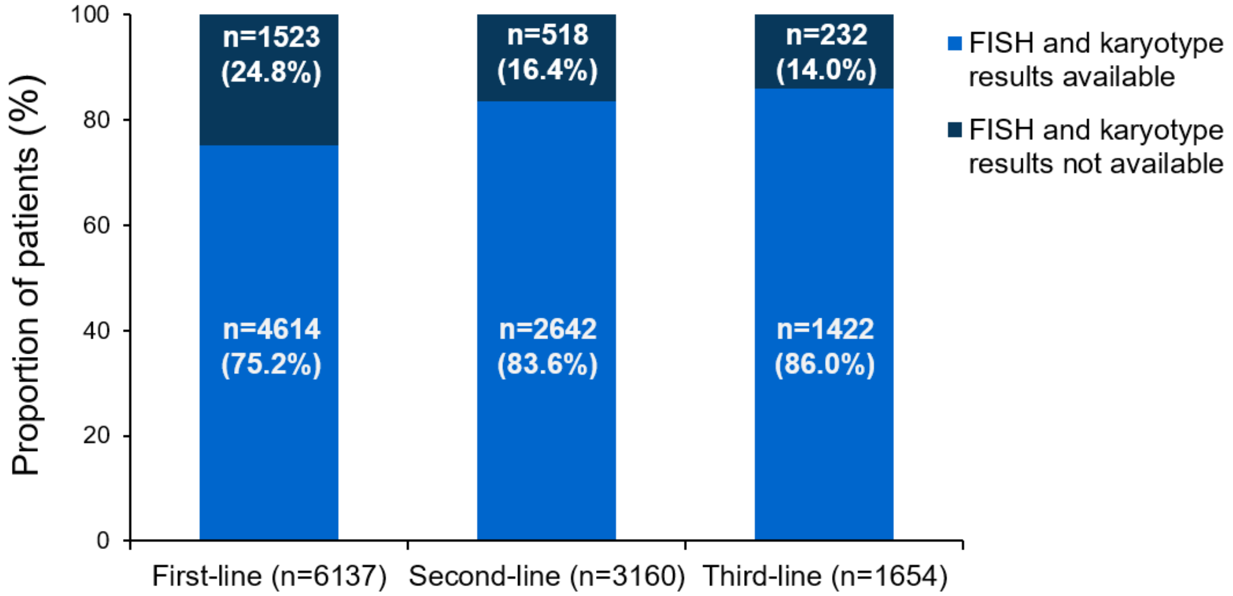

FISH fluorescence in situ hybridization.

Fig. S3 Sensitivity analysis. Treatment patterns (A) and Kaplan–Meier curves for TTNT (B), and OS (C) for patients aged <70 vs ≥70 years.

A

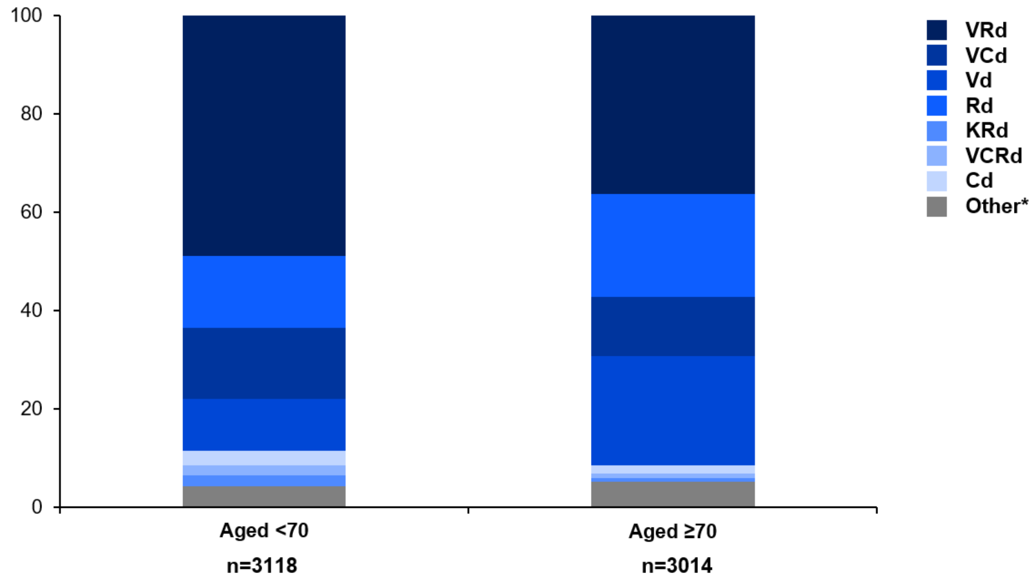

B

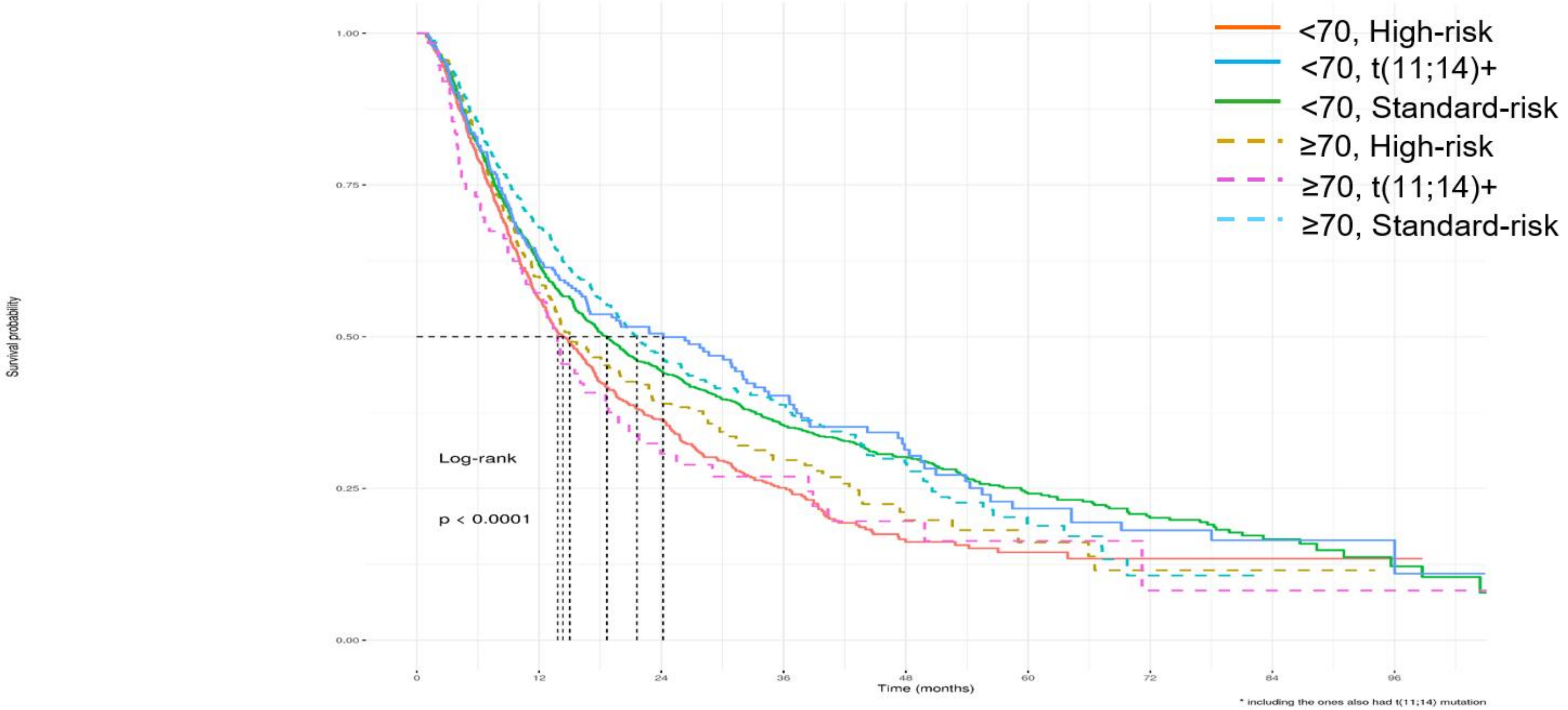

Number at risk: n (%)

Strata	0	12	24	36	48	60	72	84	96
riskgroup=Any high risk*, indexagecat=below 75 years	1173 (100)	443 (38)	208 (18)	96 (8)	37 (3)	17 (1)	9 (1)	3 (0)	1 (0)
riskgroup=Any high risk*, indexagecat=over 75 years	451 (100)	163 (36)	72 (16)	35 (8)	15 (3)	7 (2)	3 (1)	1 (0)	0 (0)
riskgroup=Standard risk, indexagecat=below 75 years	1837 (100)	866 (47)	474 (26)	290 (16)	188 (10)	99 (5)	63 (3)	25 (1)	8 (0)
riskgroup=Standard risk, indexagecat=over 75 years	707 (100)	314 (44)	155 (22)	92 (13)	42 (6)	13 (2)	4 (1)	0 (0)	0 (0)
riskgroup=t(11;14) no other h-risk, indexagecat=below 75 years	320 (100)	154 (48)	88 (28)	56 (18)	32 (10)	19 (6)	14 (4)	9 (3)	3 (1)
riskgroup=t(11;14) no other h-risk, indexagecat=over 75 years	126 (100)	41 (33)	18 (14)	12 (10)	6 (5)	3 (2)	1 (1)	1 (1)	1 (1)

Time (months)

* including the ones also had t(11;14) mutation

C

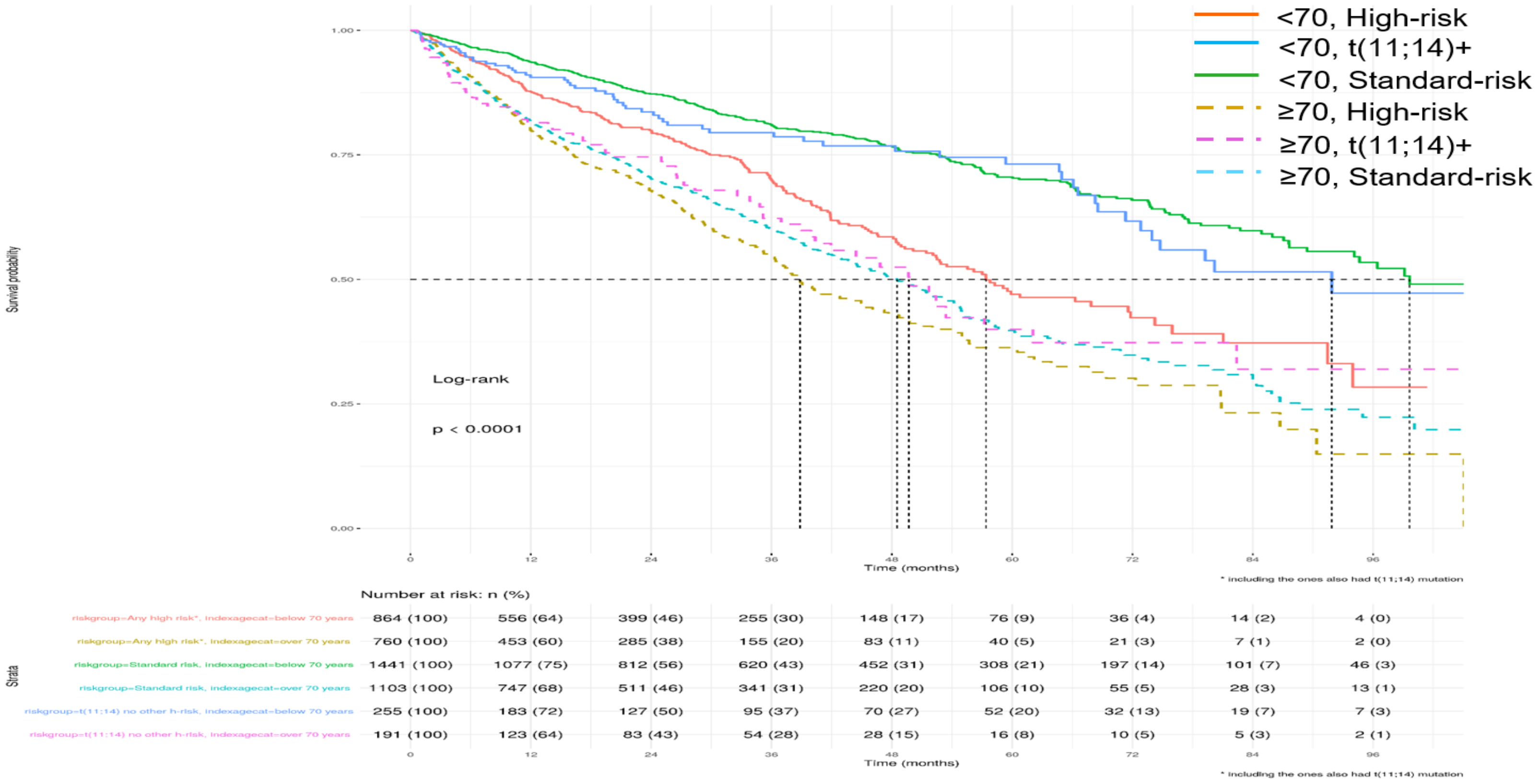

*Represents all other therapies, where <1% of patients receive a particular treatment regimen.

C cyclophosphamide, *d* dexamethasone, *del* deletion, *K* carfilzomib, *OS* overall survival, *R* lenalidomide, *t* translocation, *TTNT* time to next treatment, *V* bortezomib.

Fig. S4 Sensitivity analysis. OS for patients with t(11;14) with or without high risk factors.

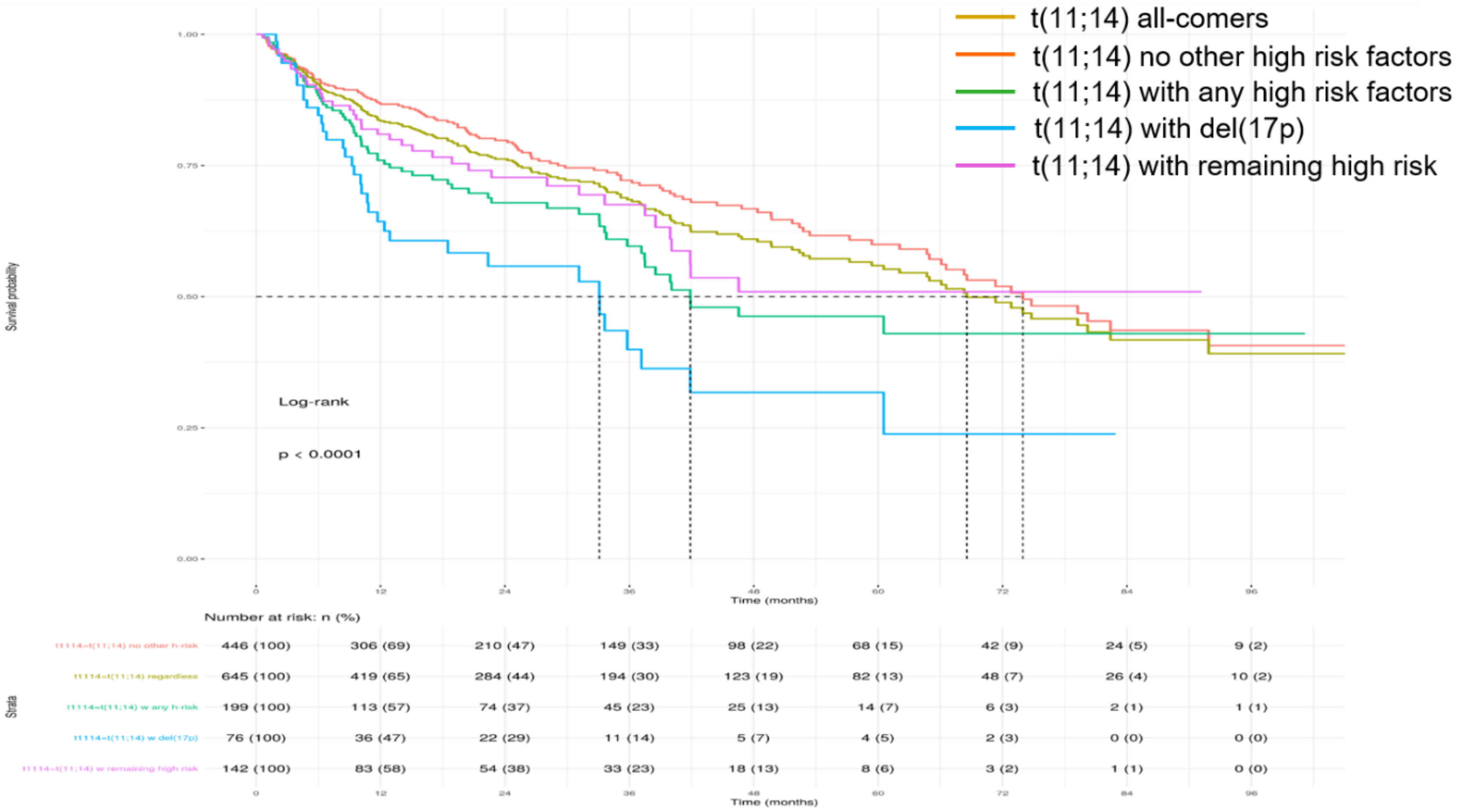

OS overall survival.

Fig. S5 Sensitivity analysis. Treatment patterns for the expanded first-line cohort (A) and Kaplan–Meier curves by risk-subgroup for TTNT (B) and OS (C).

A

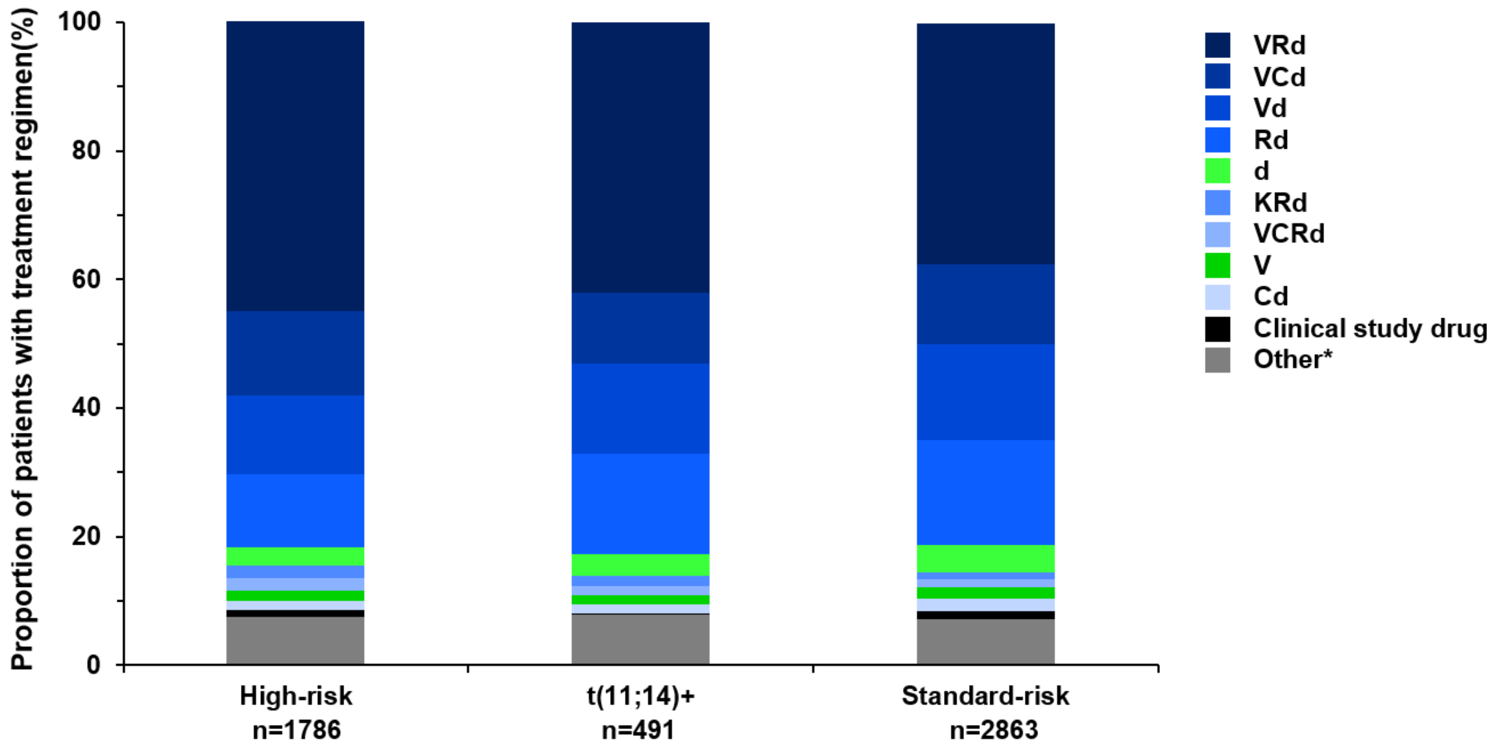

B

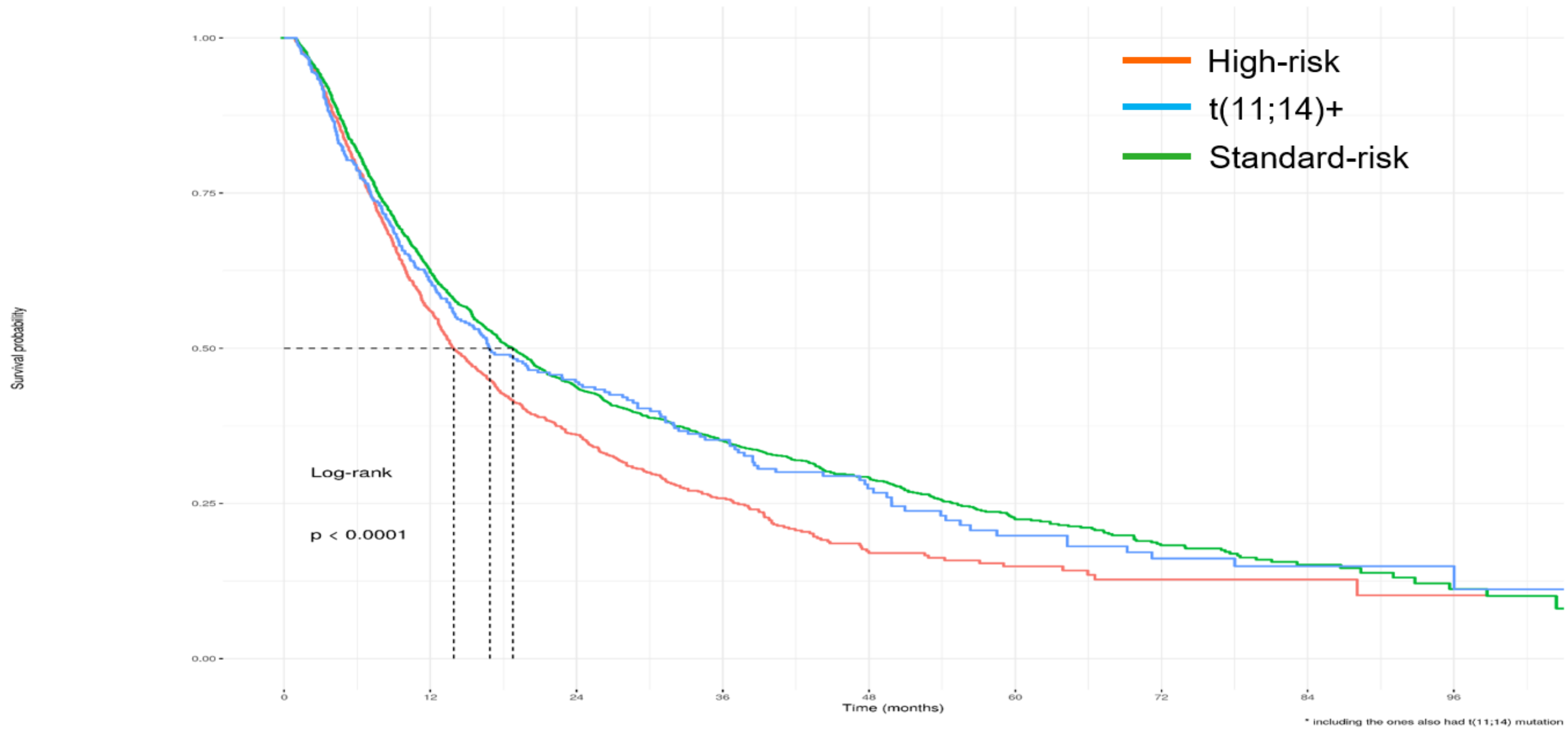

Number at risk: n (%)

Strata	0	12	24	36	48	60	72	84	96
riskgroup=Any high risk*	1786 (100)	650 (36)	300 (17)	140 (8)	54 (3)	25 (1)	13 (1)	5 (0)	1 (0)
riskgroup=Standard risk	2862 (100)	1296 (45)	687 (24)	419 (15)	255 (9)	126 (4)	77 (3)	30 (1)	12 (0)
riskgroup=t(11;14) no other h-risk	491 (100)	209 (43)	112 (23)	70 (14)	40 (8)	23 (5)	16 (3)	10 (2)	4 (1)

Time (months)

* including the ones also had t(11;14) mutation

C

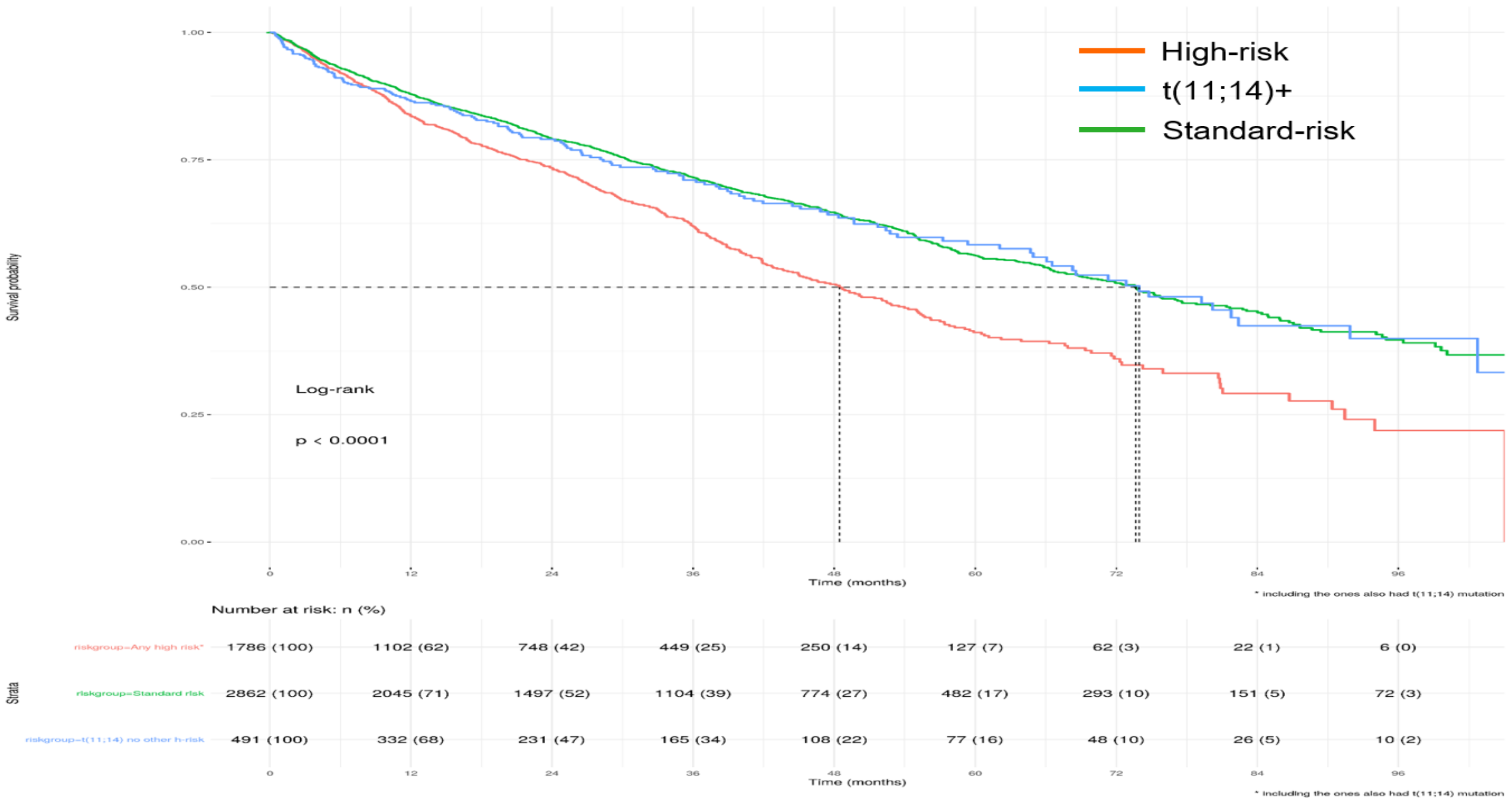

*Represents all other therapies, where <1% of patients receive a particular treatment regimen.

C cyclophosphamide, *d* dexamethasone, *K* carfilzomib, *OS* overall survival, *R* lenalidomide, *TTNT* time to next treatment, *V* bortezomib.
